# Delays in the diagnosis and treatment of tuberculosis patients in Vietnam: a cross-sectional study

**DOI:** 10.1186/1471-2458-7-110

**Published:** 2007-06-13

**Authors:** Nguyen T Huong, Marleen Vree, Bui D Duong, Vu T Khanh, Vu T Loan, Nguyen V Co, Martien W Borgdorff, Frank G Cobelens

**Affiliations:** 1 National Hospital of Tuberculosis and Respiratory Diseases, Hanoi, Vietnam; 2 KNCV Tuberculosis Foundation, The Hague, The Netherlands; 3 Center for Infection and Immunity Amsterdam, Academic Medical Center, University of Amsterdam, The Netherlands

## Abstract

**Background:**

Treatment delay is an important indicator of access to tuberculosis diagnosis and treatment. Analyses of patient delay (i.e. time interval between onset of symptoms and first consultation of a health care provider) and health care delay (i.e. time interval between first consultation and start of treatment) can inform policies to improve access. This study assesses the patient, health care provider and total delay in diagnosis and treatment of new smear-positive pulmonary tuberculosis patients, and the risk factors for long delay, in Vietnam.

**Methods:**

A cross-sectional survey of new patients treated by the National Tuberculosis Control Programme was conducted in 70 randomly selected districts in Vietnam. All consecutively registered patients in one quarter of 2002 were interviewed using a pre-coded structured questionnaire.

**Results:**

Median (range) delay was 4 weeks (1–48) for total, 3 (1–48) weeks for patient and 1 (0–25) week for health care delay. Patients with long total delay (≥ 12 weeks, 15%) accounted for 49% of the cumulative number of delay-weeks. Independent risk factors (p < 0.05) for long total delay were female sex, middle age, remote setting, residence in the northern or central area, and initial visit to the private sector. For long patient delay (≥ 6 weeks) this was female sex, belonging to an ethnic minority, and living at > 5 km distance from a health facility or in the northern area. For long health care delay (≥ 6 weeks) this was urban setting, residence in the central area and initial visit to a communal health post, TB hospital or the private sector.

**Conclusion:**

Analyses of patient and treatment delays can indicate target groups and areas for health education and strengthening of the referral system, in particular between the private sector and the NTP.

## Background

Delays in diagnosis and treatment of tuberculosis (TB) are common. They may reflect patient delays in seeking care, health care provider delays in making the diagnosis and starting treatment, or both [1-7].

Diagnostic delay may result in more advanced and severe disease, higher mortality and sustained spread of *M. tuberculosis *in the community as untreated persons continue to transmit the infection to others [8,9]. Information on diagnostic delay and its trend over time is thus important for evaluation and improvement of TB control programmes.

Distributions of diagnostic delays tend to be skewed: they are relatively short for most TB patients, but very long for some [3,4]. Patients with long diagnostic delays may therefore contribute disproportionately to the cumulative delay of all patients and thereby, potentially, to TB transmission. Prevention of these long delays could therefore be more effective in curbing TB transmission than further reduction of the median delay among all patients. Therefore, it is also important to know the risk profile of patients with long diagnostic delays.

Vietnam is among the 22 countries with a high TB burden. In 2002, the National Tuberculosis Control Program (NTP) detected 95,044 TB cases (118 per 100,000 population), including 56,811 (71/100,000) new smear-positive cases [10,11]. From 1997 onwards, the estimated case detection rate of the NTP has been above the WHO target of 70%. One would thus expect diagnostic delays to be relatively short, but no nationally representative data are available. A study in 4 provinces in Vietnam in 1996 showed a mean total delay among TB patients of 12 weeks [12]; only small-scale studies of delay have been published since [13-15]. In these studies, women had longer delays than men, and more often had visited less qualified health care providers before a diagnosis of TB was made [12-15]. In the NTP, the district is the key level for diagnosis and treatment. Patients with TB symptoms can choose any (private or public) health care provider or go directly to the district TB unit (DTU). Referral to the DTU by private health care providers tends to be limited [16]. The growth of the private health sector may thus affect diagnostic delays [17,18].

We performed a nation-wide study of diagnostic delays in 70 randomly selected district TB units, with the aims of assessing the extent of delay in case-finding and treatment among TB patients diagnosed within the NTP, and identifying risk factors associated with long delay, in particular related to choice of initial health care provider.

### Study population and methods

Eligible were all patients who were registered with newly diagnosed smear-positive pulmonary TB in the third quarter of 2002 in 70 district TB units. These districts had been sampled for a national TB prevalence survey. Sampling was with probability proportional to population size after stratification by area, so that 20 urban, 20 remote and 30 rural districts were selected. Excluded were patients with permanent or temporary residence outside the district.

Patient delay was defined as the period between the onset of cough and the patient's first attendance of a health care facility because of this cough. Health care provider (HCP) delay was defined as the period between the patient's first attendance of a health care facility with cough, and the onset of treatment. Treatment delay was defined as the period between diagnosis of smear-positive pulmonary TB and the onset of treatment. Total delay was combined patient and HCP delay. Delay periods were recorded in weeks, rounded to the nearest integer value. Long patient delays and health care provider delays were defined as 6 weeks or more and long total delay as 12 weeks or more.

Patients were interviewed using a pre-coded structured questionnaire including demographic variables (age, sex, ethnicity, education and distance from the patient's house to the first health care provider); time period between onset of cough and first contact with health care provider; time period between onset of cough and start of treatment; date of diagnosis; starting date of treatment. The district TB coordinators were trained to perform interviews and interviewed patients within 2 weeks of treatment registration. The study protocol was approved by the Scientific and Ethical Board of the National Hospital (then: Institute) of Tuberculosis and Respiratory Diseases, Hanoi.

Data were double entered using Epi Info v6; inconsistencies were checked against the raw data. Data were analysed using Stata v8. The chi-square tests were used to compare differences in proportions in total delay between subgroups. Because delays had non-normal distributions, Wilcoxon Rank Sum and Kruskal-Wallis tests were use to test for significance. Mean delays are nonetheless reported for comparison with other studies. Uni- and multivariate logistic regression analysis was used to identify risk factors that were associated with long delay. We tested for significance using the likelihood ratio test for comparing the model likelihood with and without the variable or interaction term of interest.

## Results

During the study period, 2,093 smear positive pulmonary TB patients were registered and interviewed. Among the subjects interviewed, 1,898 (92%) patients were interviewed within one week after starting treatment. Of the study subjects, 1,491 (71%) were male. The male-to-female ratio (2.5:1) reflected the male-to-female ratio of notified smear positive pulmonary patients in Vietnam in 2002 (2.4:1, NTP unpublished data). Information on total, patient and HCP delay was available for 2,069 (99%), 2,075 (99%) and 2,034 patients (97%), respectively.

Mean total delay was 7.5 weeks with a median of 4 weeks (range: 1–48). Total delay tended to be longer among women and with increasing age, as well as in the northern part of the country, in rural and remote districts settings, among ethnic minority groups and among patients who presented initially to a pharmacy or a communal health post (Table 1).

**Table 1:** Characteristics of, and total delays among 2,093 new smear-positive pulmonary tuberculosis patients in Vietnam

	N (%)	Total delay in weeks			Percentage due to patient delay
		Mean (95% CI)	P-value	Median (25–75 percentiles)	
Time (weeks)		7.5 (6.8–7.5)		4 (3–8)	62.7%
Sex			< 0.001		
Men	1491 (71.4)	7.1 (6.4–7.8)		4 (3–8)	62.0%
Women	596 (28.6)	8.4 (7.4–9.4)		5 (4–9)	66.7%
Age (years)			0.001		
0–24	187 (9.1)	5.6 (5.0–6.3)		4 (3–8)	60.7%
25–34	328 (15.9)	6.3 (5.4-7.1)		4 (3–8)	61.9%
35–44	445 (21.7)	8.1 (6.1–10.1)		4 (3–8)	58.0%
45–54	378 (18.4)	7.5 (6.5–8.5)		4 (3–8)	68.0%
55–64	251 (12.2)	8.1 (6.7–9.4)		5 (3–9)	66.7%
65+	464 (22.6)	8.2 (6.9–9.4)		5 (4–8)	64.6%
Ethnicity			0.048		
Viet	1944 (94.0)	7.4 (6.8–8.0)		4 (3–8)	62.2%
Ethnic minority	124 (6.0)	8.4 (6.7–10.0)		5 (4–9)	86.9%
Education level			0.013		
Low	895 (42.9)	7.5 (6.8–8.2)		4 (3–8)	65.3%
Middle	793 (38.1)	7.5 (6.8–8.2)		5 (3–8)	64.0%
High	396 (19.0)	7.3 (5.1–9.5)		4 (3–8)	57.5%
Distance from health facility			0.373		
0–5 km	1504 (73.4)	7.5 (6.7–8.2)		4 (3–8)	57.3%
More than 5 km	544 (26.6)	7.2 (6.4–8.1)		4 (3–8)	77.8%
Health care provider visited initially because of cough			< 0.001		
District health center	339 (16.3)	6.6 (5.9–7.2)		4 (3–8)	89.4%
Commune health post	620 (29.9)	9.1 (6.3–11.9)		5 (4–8)	67.0%
Public hospital	360 (17.4)	5.6 (4.9–6.3)		4 (2–6)	80.4%
Pharmacy	326 (15.7)	9.0 (8.0–9.9)		6 (4–12)	21.1%
Private physician	339 (16.3)	7.2 (6.2–8.2)		4 (3–8)	45.8%
Traditional healer	32 (1.5)	6.7 (5.0–8.4)		6 (4–8)	58.2%
Other	59 (2.8)	12.7 (6.5–18.8)		4 (3–10)	78.7%
Area			< 0.001		
Rural	1029 (49.2)	8.1 (7.4–8.9)		5 (4–8)	65.4%
Urban	808 (38.6)	5.8 (5.4–6.2)		4 (3–7)	56.9%
Remote	256 (12.2)	9.9 (6.4–13.3)		5 (3–9)	71.7%
Region (n = 2,087)			< 0.001		
South	1316 (62.9)	5.7 (5.3–6.0)		4 (3–6)	52.6%
North	567 (27.1)	11.2 (9.3–13.1)		6 (4–10)	81.3%
Central	210 (10.0)	8.6 (7.2–10.0)		5 (4–9)	44.2%

Mean patient delay was 4.7 weeks, with a median of 3 weeks (range: 1–48). Men reported earlier with TB symptoms than women (4.4 and 5.6 weeks respectively, p < 0.005). Increasing age and increasing distance between the patient's home and the health care facility visited initially were associated with longer patient delay (p < 0.05). Mean patient delay was longer for patients who initially visited public health facilities such as communal health posts (6.1 weeks), district health centers (5.9 weeks) or hospitals (4.5 weeks) than for patients who first visited traditional healers (3.9 weeks), private practitioners (3.3 weeks) or pharmacies (1.9 weeks) (p < 0.005). Patients delays were also longer in remote (7.1 weeks) or rural settings compared to urban settings (5.3 weeks, p < 0.005), and in the northern region (9.1 weeks, p < 0.005).

Patient delay accounted for 63% of total delay overall. This proportion was lowest for patients who initially visited a pharmacy (21%) or a private practitioner (46%), or who lived in the central region (44%). The proportion of patient delay out of total delay was highest for patients who initially visited a district health center (89%) or public hospital (80%), patients who belonged to ethnic minorities (87%), and patients who lived in the northern region (81%) (Table 1).

Mean health care provider (HCP) delay was 2.8 weeks with a median of 1 week (range: 0–25). HCP delays were longer for women (2.9 weeks, p < 0.005) and for patients aged 34–45 years (3.5 weeks) or 65 years or more (3.1 weeks). HCP delays were also longer for patients with a high level of education (3.2 weeks), or who visited initially the private sector (p < 0.001), as well as for patients living at more than 5 km distance from the health facility (p < 0.005), patients living in rural areas (p < 0.001), and patients in the central region (p < 0.001)

Long total delay was observed for 305 patients (15%, 95% CI 13%–16%), long patient delay for 434 (21%, 95% CI 19%–23%), and long HCP delay for 248 (12%, 95% CI 11%–14%). Of 1,731 patients with a total delay ≤ 12 weeks, 99 (6%) reported long HCP delay and 239 (14%) long patient delay (p < 0.001). Of 303 patient with long total delay, 128 (42%) reported long HCP delay, 154 (51%) reported long patient delay, and 21 (7%) reported both (p < 0.001). Thus, patient delay contributed most to long total delay.

In a logistic regression model, long total delay was significantly associated with initial visit to a pharmacy (adjusted odds ratio, aOR 4.3) or a private practitioner (aOR 1.7). In addition, it was associated with middle age (aOR 1.8 for 34–44 years and aOR 2.1 for 44–54 years), with living in remote areas (aOR 1.6) and with living in the central or northern compared to the southern region (aOR 3.8 and aOR 2.9 respectively; Table 2).

**Table 2:** Univariate and multivariate analysis of risk factors associated with long delay among new smear-positive pulmonary tuberculosis patients in Vietnam

	Long total delay			Long patient delay			Long HCP delay		
	N (%)	OR	aOR* (95% CI)	N (%)	OR	aOR* (95% CI)	N (%)	OR	aOR* (95% CI)
Sex									
Men	189/1447 (12.8)	1	1	285/1,476 (19.3)	1	1	169/1449 (11.7)	1	1
Women	115/586 (19.6)	1.7	1.6 (1.3–2.2)	149/593 (25.1)	1.4	1.3 (1.0–1.7)	78/579 (13.5)	1.2	1.2 (0.8–1.7)
Age (years)									
0–24	18/186 (9.7)	1	1	30/186 (16.1)	1	1	20/184 (10.9)	1	1
25–34	35/323 (10.8)	1.1	1.2 (0.6–2.2)	60/326 (18.4)	1.2	1.1 (0.7–1.9)	32/316 (10.1)	0.9	0.9 (0.5–1.8)
35–44	72/443 (16.3)	1.8	1.8 (1.0–3.2)	81/439 (18.5)	1.2	1.1 (0.6–1.8)	61/434 (14.1)	1.3	1.2 (0.6–2.2)
45–54	66/374 (17.7)	2.0	2.1 (1.2–3.8)	81/375 (21.6)	1.4	1.2 (0.7–2.0)	44/369 (11.9)	1.1	1.2 (0.6–2.3)
55–64	38/249 (15.3)	1.7	1.4 (0.7–2.7)	61/251 (24.3)	1.7	1.1 (0.7–2.0)	24/246 (9.8)	0.9	0.9 (0.4–1.9)
65+	73/455 (16.0)	1.8	0.9 (0.8–2.6)	113/458 (24.7)	1.7	1.2 (0.7–2.0)	65/446 (14.6)	1.4	1.6 (0.8–2.9)
Health care provider visited initially because of cough									
District health center	78/617 (12.6)	1	1	197/620 (31.8)	1	1	7/611 (1.2)	1	1
Commune health post	46/333 (13.8)	1.0	0.9 (0.6–1.4)	92/339 (27.1)	0.8	0.6 (0.4–0.8)	22/330 (6.7)	5.8	6.3 (2.6–15.2)
Public hospital	39/357 (10.9)	0.8	1.1 (0.7–1.7)	77/260 (21.4)	0.6	0.8 (0.6–1.2)	15/355 (4.2)	3.2	3.3 (1.3–8.3)
Pharmacy	81/322 (25.2)	2.3	4.3 (2.9–6.5)	18/326 (5.5)	0.1	0.2 (0.1–0.4)	135/321 (42.1)	59.0	66.0 (29.6–147.3)
Private physician	43/337 (12.8)	1.0	1.7 (1.1–2.6)	31/339 (9.1)	0.2	0.4 (0.2–0.6)	54/331 (16.3)	15.6	16.1 (7.1–36.6)
Traditional healer	5/31 (16.1)	1.3	2.0 (0.7–5.7)	5/32 (15.6)	0.4	0.5 (0.2–1.4)	8/29 (27.6)	31.8	41.5 (13.3–129.3)
Other	13/44 (22.8)	1.9	2.3 (1.1–4.9)	14/59 (23.7)	0.7	0.8 (0.4–1.5)	7/57 (12.3)	9.9	10.6 (3.4–33.3)
Area									
Rural	159/1016 (15.7)	1	1	245/1023 (24.0)	1	1	120/1002 (12.0)	1	1
Urban	99/800 (12.4)	0.8	1.0 (0.7–1.4)	109/798 (13.7)	0.5	0.7 (0.5–0.9)	111/673 (14.2)	1.2	1.6 (1.1–2.2)
Remote	47/253 (18.6)	1.3	1.6 (1.1–2.3)	80/254 (31.5)	1.5	1.2 (0.8–1.7)	17/248 (6.9)	0.5	1.4 (0.8–2.6)
Region									
South	134/1302 (10.3)	1	1	143/1305 (11.0)	1	1	175/1280 (13.7)	1	1
North	129/558 (23.1)	2.6	3.8 (2.7–5.4)	261/561 (46.5)	7.4	4.9 (3.7–6.5)	28/547 (5.1)	0.3	1.0 (0.6–1.6)
Central	42/209 (20.1)	2.3	2.9 (1.9–4.4)	20/209 (14.4)	1.5	1.4 (0.9–2.2)	45/207 (21.7)	1.8	3.0 (1.9–4.8)
Total	305/2069 (14.7)			434/2075 (20.9)			248/2034 (12.2)		

Long total delay: ≥ 12 weeks. Long patient delay: ≥ 6 weeks. Long HCP delay: ≥ 6 weeks. * Adjusted for variables of sex, age, initial visit, area and region in the model. OR: Odds ratio; aOR: adjusted odds ratio. 95% CI: 95% confident interval. HCP: Health care provider.

The logistic regression models for long patient delay and HCP delay showed opposite associations with initial health care provider (Table 2). Long patient delay was more common among patients who initially visited a public provider, whereas long HCP delay was substantially more common among patients who initially visited a private provider. Compared to the district health center as the initial provider, the relative risks were 66 for pharmacy, 42 for traditional healer, and 16 for private practitioner. Other independent risk factors for long patient delay were female sex, belonging to an ethnic minority, living at more than 5 km distance from the DTU, and residence in the northern region. Other independent risk factors for long HCP delay were living at more than 5 km distance from the DTU and residence in the central region (Table 2).

In each of the logistic regression models there was significant interaction related to the initial HCP. For total delay, this differed significantly by region. Compared to a public health facility as first provider, the risk of long total delay when the first provider was a pharmacy, private practitioner, traditional healer or other private provider was 3.9 times increased (OR 3.9, 95% CI 2.6–6.1) in the south and 2.8 times (OR 2.8, 95% CI 1.3–6.1) in the center, but only 1.2 times in the north (OR 1.2, 95% CI 0.7–2.3; likelihood ratio test for interaction: p < 0.01). The risk of long patient delay if the first provider was private rather than public was reduced by half among men (OR 0.5, 95% CI 0.4–0.8), but to one third among women (OR 0.3, 95% CI 0.1–0.5; p < 0.05). The risk of long HCP delay if the first provider was private rather than public was 10.5 times increased in rural districts (OR 10.5, 95% CI 5.9–18.6) and 16.8 times in urban districts (OR 16.8, 95% CI 9.0–31.7), but only 3.9 times in remote districts (OR 3.9, 95% CI 1.2–2.5; p < 0.05).

**Figure 1:**
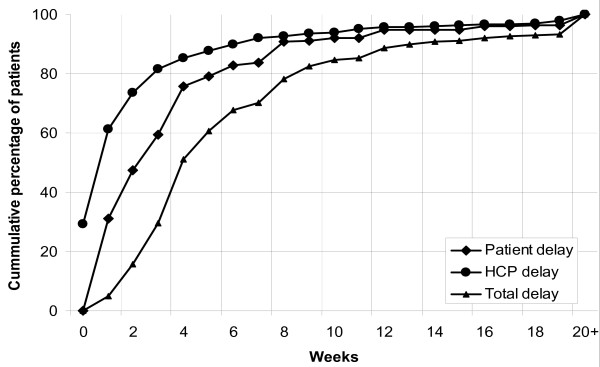
Cumulative proportion of patient, health care provider and total delay among new smear-positive tuberculosis patients.

**Figure 2:**
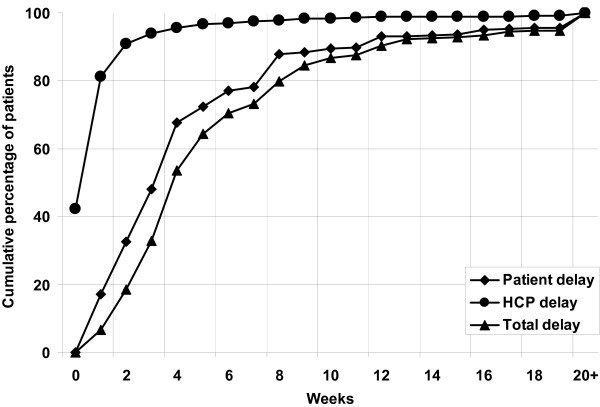
Cumulative proportion of patient, health care provider and total delay among new smear-positive tuberculosis patients who initially visited public health care providers.

**Figure 3:**
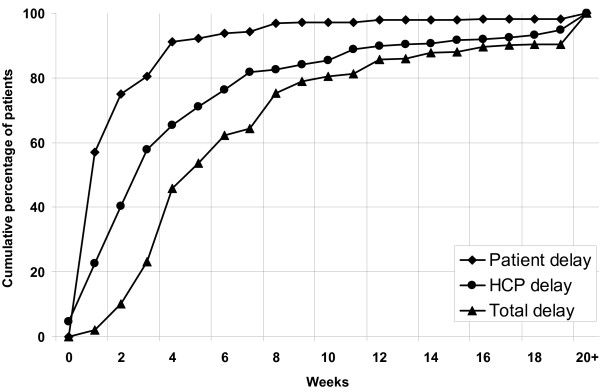
Cumulative proportion of patient, health care provider and total delay among new smear-positive tuberculosis patients who initially visited private health care providers.

The associations with initial provider were further analyzed by comparing the distribution of delay periods (Figures 1, 2 and 3). Eighty-eight percent of patients who initially visited a public provider had been detected and started treatment within 12 weeks, compared to 81% of patients who initially visited a private provider (p < 0.001). Of the patients who initially visited a public provider, 72% had reported with symptoms within 6 weeks of onset, compared to 92% of patients who initially visited a private provider (p < 0.001). Within 6 weeks of the initial visit, 97% of patients had started TB treatment if the initial provider had been public, compared to 71% if the initial provider had been private (p < 0.001). Overall, patients with long total delay accounted for 49% of the cumulative number of delay-weeks of all patients. This was 62% both for long patient and for long HCP delay.

## Discussion

This study showed an overall mean time from onset of cough to TB treatment for new smear-positive pulmonary TB patients in Vietnam of 7.5 weeks. This is almost half less than the delay reported from 4 provinces in Vietnam in 1996 (13.3 weeks) [12], and much lower than the delay reported from other high-prevalence countries such as Nepal [19], South Africa [20], Tanzania [2], Ethiopia [3,4] and Thailand [7], where it ranged from 9 to 23 weeks.

Patient delay (on average 4.7 weeks) contributed more than HCP delay (on average 2.8 weeks) to the total delay in our study. In 1996 mean patient delay was 5.8 weeks and mean HCP delay was 6.1 weeks [12]. Although the study in 1996 measured delay by onset of any TB symptom instead of just cough, and was not representative for Vietnam, the data suggest a decrease in total delay since 1996 that is primarily due to a decrease in HCP delay.

The short delay found in our study may be explained by the development of the general health care and TB control network in Vietnam over the past years [21,22]. This may have impacted both on patient and on HCP delays. Access of the poor to basic health services has been improved by providing free health cards, user fee exemption, reimbursement of travel cost and other enablers [21,22]. Knowledge of commune health staff and volunteers at village level also has improved over the recent years. A community health network enhances detection and referral of patients who are suspect of having TB to the appropriate level for diagnosis [23]. Health information campaigns may have raised the awareness of the community about TB symptoms and the need for timely diagnosis [23].

We found clear associations between delay and the type of health care provider first visited by the patient because of cough. Long patient delays were most frequent if this had been the district health centre, and least frequent if this had been a private provider. Long HCP delays were however more frequent among patients who visited a private provider first, as were long total delays. These findings are in accordance with data from Ho Chi Minh City, where, compared to public providers, relative risks for long HCP delay were 5.5 for private pharmacies as the first provider, and 2.1 for private physicians as the first provider [13]. Thus, although initial visits to private health services tended to be earlier after the onset of cough than initial visits to public health services, the time until diagnosis of TB tended to be longer, to the net effect of longer delays if the initial provider had been private. This suggests that referral from the private sector is an important problem. One-third of all patients in our study had initially visited a private provider, although this proportion was higher in the southern (44%) and central (37%) than in the northern region (8%). Also, the observed interaction indicates that referral from private providers is less of a problem in the north and in remote settings. Education on diagnosis of TB of private health care providers and pharmacies may help prevent long health care provider delays, as may implementation of public-private mix DOTS projects in urban and rural areas, in particular in the southern and central regions. The growth of the private sector in Vietnam and its impact on diagnostic delay warrant regular monitoring of these delays.

The 21% of patients who had patient delays of more than 6 weeks accounted for 61% of the cumulative patient delay period, and the 13% of patients who had HCP delay of more than 6 weeks accounted for 62% of the cumulative HCP delay. It supports our hypothesis that efforts should not be put in further reduction of total median delay, but in prevention of long delays, since they contribute most to the cumulative delay of all patients and thereby to transmission.

The sex difference in delay is small and similar to the difference found by previous studies [12,14,15,24-26]. These longer delays among women, as well as the low proportion of women among patients in this study and among notified TB patients in Vietnam in general, may indicate lower access to diagnosis and treatment of TB for women compared to men [12,25,26]. However, biological differences between men and women with respect to TB incidence and sensitivity of smear examination may play a role as well [24,27]. Analysis of model interactions suggests that, although women tend to report with cough to private providers earlier than men, this does not result in shorter overall delays, supporting earlier findings that health care providers visited by women tend to be less qualified [24,25]. These analyses showed other risk factors for long delay that may help target interventions, such as belonging to an ethnic minority, living in particular geographic areas, middle age and female sex.

There were several limitations to our study. First, the reported duration of symptoms is based on the patients' recall and interpretation. Recall bias is thus a serious threat to the estimates of delay and actions taken. Unfortunately there is no feasible method which can deal with this problem. Second, selection of patients might be biased. Some patients diagnosed within the NTP may have been referred to other facilities or have sought treatment in the private sector before they were registered. These patients were not included in our study. Third, both health workers and patients can contribute to long HCP delay: patients can delay the start of treatment as well as the health worker, e.g. for fear of stigmatization or reluctance to tell employers [28]. Finally, the observed association of long delay with private health care provider does not necessarily imply causation. Patients may have been more "at risk" of visiting a private practitioner simply because more time elapsed between onset of symptoms and diagnosis at the TB unit.

## Conclusion

On average, total, patient and health care provider delays were short in this nation-wide study of TB patients in Vietnam. Nonetheless, 15% of patients reported total delays ≥ 12 weeks, and accounted for half of the cumulative number of delay-weeks. Initial visit to the private sector had a considerable impact on the length of delays but with distinct regional differences across the country. Efforts should be made to further reduce diagnostic delays by improving the referral from the private to the public health sector.

## Abbreviations

aOR adjusted odds ratio

CI confidence interval

DTU district tuberculosis unit

HCP health care provider

NTP National Tuberculosis Control Programme (Vietnam)

OR odds ratio

TB tuberculosis

WHO World Health Organization

## Competing interests

The author(s) declare that they have no competing interests.

## Authors' contributions

NTH: conception and design of the study, data collection, analysis and interpretation, writing of the manuscript. MV: assisted in data analysis and writing of the manuscript. BDD: conception and design of the study, data collection, analysis and interpretation, critical review of the manuscript. VTK: design of the study, data collection, critical review of the manuscript. VTL: design of the study, data collection, critical review of the manuscript. NVC: conception and design of the study, critical review of the manuscript. MB: design of the study, critical review of the manuscript. FC: supervision of data collection and management, assisted in data analysis, critical review of the manuscript. All authors read and approved the final manuscript

## Pre-publication history

The pre-publication history for this paper can be accessed here:


